# Comparative Evaluation of the Digital Workflow and Conventional Method in Manufacturing Complete Removal Prostheses

**DOI:** 10.3390/ma16216955

**Published:** 2023-10-30

**Authors:** Sara Dib Zakkour, Juan Dib Zakkour, Yasmina Guadilla, Javier Montero, Abraham Dib

**Affiliations:** 1 Dental Clinic Dib Bucodental S.L., 37770 Guijuelo, Spain; sdib@usal.es; 2 Dental Clinic of the Faculty of Medicine, University of Salamanca, 37008 Salamanca, Spain; yguadilla@usal.es (Y.G.); javimont@usal.es (J.M.); ibrahimdib@usal.es (A.D.)

**Keywords:** digital, removable complete dental prosthesis, CAD/CAM, complete denture

## Abstract

The aging population in developed countries has increased the number of edentulous patients and, therefore, the need for prosthetic rehabilitation to improve their quality of life. Complete dentures are the main treatment option in these cases. The use of CAD/CAM (Computer Aided Design/Computer Aided Manufacturing) in dentistry has improved clinical protocols and outcomes, achieving a reduction in work time and economic costs for the patients. The main objective of this review was to compare the characteristics of conventional and digital dentures, attempting to determine whether the use of new technologies represents an improvement in the properties of removable complete dentures. A bibliographic review was carried out in the PubMed/MEDLINE, Cochrane Library, Scielo, and Embase databases. With the initial search, 157 articles were obtained. After applying the inclusion and exclusion criteria, 64 publications were selected for this bibliographic review. The different conclusions of the studies consulted were compared regarding fit and retention, fracture resistance, surface roughness, biocompatibility, and aesthetics, taking into account the different methods of prostheses fabrication. In general, digital prostheses have shown better mechanical properties and, consequently, better biocompatibility and aesthetics than conventional prostheses. However, the obtained results were very heterogeneous, preventing a supported conclusion.

## 1. Introduction

Currently, developed countries are facing significant population aging, leading to an increase in the prevalence of partially or completely edentulous patients, resulting in the need for prosthetic treatments [1,2,3]. The conventional complete denture is considered the primary treatment option for these patients. Implant-supported or implant-retained prostheses are good alternatives, but they might be contraindicated or inaccessible for some patients, due to economic reasons, systemic conditions that contraindicate surgery, a complete lack of oral hygiene by the patient, anatomical constraints such as insufficient bone quantity or quality, or head and neck radiation therapy or chemotherapy, among others [4,5]. These situations make conventional complete dentures a valid option for most edentulous patients [2,3]. Over the past decades, with the rapid advancement of science and new technologies, the use of digital methods in dentistry, including the field of complete dentures, has significantly increased [6].

The conventional protocol for fabricating a mucosa-supported prosthesis involves a lengthy sequence of clinical and laboratory steps, which can result in a relatively long time period for completion. The polymerization shrinkage of polymethyl methacrylate (PMMA) and the difficulty in duplicating the prosthesis in case of a need to revert to earlier manufacturing stages can be considered the main drawbacks of the technique [7,8,9,10,11].

The use of CAD/CAM (computer-aided design/computer-aided manufacturing) in dentistry began in the early 1980s [3]. Currently, CAD/CAM is more advanced compared to conventional techniques [12]. The implementation of CAD/CAM in dental prosthesis has resulted in easier clinical protocols, the use of material with better properties, improved fit and retention of prostheses, a reduction in chairside and laboratory working time, and overall cost reduction. However, adopting this technology requires a learning curve and the digitalization of clinical procedures. The combined use of optical scanner and conventional techniques has shown success, while fully digital workflows still require further studies [13,14,15,16,17]. Within digital fabrication of complete prostheses and other elements, two techniques can be distinguished [3,7,18]:

1. Subtractive or milling technique. It utilizes pre-polymerized PMMA discs that are milled under high pressure and controlled conditions. Recent research has shown that milled resins have better mechanical and surface properties (greater color stability, reduced bacterial colonization, and lower monomer release) than thermopolymerizable resins [19]. It is commonly used for fabricating fixed dental prostheses, inlays, onlays, veneers, or crowns [20,21,22].
2. Additive or 3D printing technique. The additive manufacturing involves layer-by-layer deposition of liquid resins on a support structure, followed by curing with visible light, ultraviolet light, heat, or laser. This process is repeated until the complete prosthesis takes the shape specified in the digital design software. The use of this technique is increasing in dentistry as it allows fabrication not only of complete dentures but also fixed prostheses, surgical guides, occlusal splints, and more [20].

Initially, most manufacturers use milling for producing complete dentures, while the additive manufacturing is mainly used for fabricating provisional prostheses, but this has been changing as the materials used in 3D printing have evolved [23,24].

Some advantages of CAD/CAM include the possibility to store patient anatomical data and prosthesis design, making it feasible to reproduce the prosthesis in case of loss or damage without repeating patient records. Additionally, CAD/CAM eliminates the polymerization shrinkage of the prosthetic base, reduces the clinician’s workload, and results in a higher strength and density of the material used [25,26,27]. The digital fabrication technique of complete dentures differs from the conventional technique, mainly in the need for impressions that capture both the internal and external surface of the prosthesis, as well as functional impressions [12]. Currently, using an intraoral scanner on completely edentulous arches does not achieve the same precision as conventional impressions, as scanning accuracy decreases with an increasing number of missing teeth. Several factors, such as the characteristics of the scanned surface, oral cavity conditions, or patient movements, can negatively impact the quality of the impression. Anatomical deviations such a high-arched palate, wide edentulous gaps, or the patient´s inability to open their mouth can also become limitations of the intraoral scanner [28,29]. Digital complete dentures can be manufactured from scratch or copying a previous denture. If the patient does not have existing dentures, irreversible hydrocolloid impressions will be taken to obtain plaster models, on which thermopolymerizable trays will be adapted. The vertical dimension (VD) will be obtained with phonetic registrations, mainly using bilabial sounds, and the occlusal vertical dimension (OVD) will be calculated from it [3,30]. The digital fabrication technique depends on the system being used.

In milling manufacturing, the prostheses are manufactured from a cylinder of acrylic resin produced under high pressure and temperature, preventing the shrinkage of the final milled prosthesis. As a result of compressing this resin, there is a decrease in monomer release, reduced porosity compared to conventional prostheses, and a reduction in *Candida albicans* adhesion to the prosthetic base. The teeth used in milled prostheses are not milled; instead, they are the same as those used in conventional prostheses [12,14,31]. The design software positions the teeth according to the desired occlusion, using a transparent guide to place the maxillary anterior teeth. At this point, the preliminary prosthesis design in sent to the dentist, who can modify it if necessary. Some design software, like the 3Shape Dental System 2013, can simulate mandibular movements (laterotrusions, protrusions, and retrusions), allowing the occlusion to be adjusted based on the registered mandibular movements. However, this step should not replace the final occlusion adjustment once the prostheses are placed in the patient´s mouth [3]. Once the teeth design is approved by the dentist, the prosthesis base and tooth sockets (simulating alveoli) are milled. Finally, the artificial teeth are chemically bonded to the base using a bonding technique that involves heat and pressure. In the conventional method, the bonding between teeth and denture base occurs during the polymerization process, while in digital prostheses, the bonding takes place after the polymerization of the base. The release of the monomer that occurs during polymerization reduces the bonding capacity of the resin. This requires the use of techniques that modify the surfaces, primarily airborne-particle abrasion or sandblasting, to strengthen the chemical bond formed through the use of appropriate adhesives [32,33,34].

To assess the quality of a removable prosthesis, studies rely on the following criteria: aesthetics (lip support and smile line), retention, stability, support, and balanced occlusion. However, patient satisfaction with the prosthesis is also crucial and remains essential in evaluating its quality [1,25].

In milled prostheses, a decrease in stability-related problems has been observed because the prosthesis is milled from a pre-polymerized acrylic resin disc [14,35]. This improvement results not only in enhanced stability but also in better retention, leading to reduced trauma and a decreased need for adjustments after insertion [14,35]. In conventional techniques, the deformation of the thermopolymerizable resin can decrease the degree of adaptation of the prosthetic base, which can be compensated by sealing the posterior part of the palate [30]. Complete dentures made from materials with low surface hardness can suffer damage from aggressive brushing, leading to plaque retention and discoloration, thereby reducing the prosthesis´ lifespan [30,36]. Surface roughness is one of the most critical factors influencing bacterial colonization of material, as well as color stability. In CAD/CAM-fabricated dentures, the bases are milled from resin discs subjected to high pressure during polymerization. This milling process produces smoother surfaces, resulting in reduced microbial adhesion compared to conventional fabrication methods [18,30]. Biocompatibility is one of the most crucial qualities to consider when selecting a material. Oral mucosa in contact with a foreign material may experience adverse reactions such as pain, hypersensitivity reactions or a burning sensation in the mouth. Therefore, assessing the material´s biocompatibility before initiating treatment is essential to ensure patient satisfaction [18]. Regarding aesthetics, dentists prefer the aesthetics of conventional dentures [35]. The aesthetics of CAD/CAM-fabricated dentures remains a limitation. Additionally, one of the advantages of conventional dentures is the prosthetist´s ability to work collaboratively with the dentist and patient, considering their preferences in the fabrication process [30].

This literature review was conducted to compare the properties of conventional and digital prostheses, aiming to determine whether the new manufacturing techniques using advances technologies result in improvements nor only in terms of cost-effectiveness and time saving but also in treatment quality and patient satisfaction. The review focused on digitally manufactured prosthetics produced mainly through milling, primarily due to a larger amount of available research in the literature. So, the results, despite including both manufacturing techniques, are primarily applicable to the subtractive technique. The specific objectives proposed were as follows:

1. A comparison of the properties of the final prosthesis, as well as the advantages and disadvantages between both conventional and CAD/CAM methods of fabricating complete mucosa-supported dentures.
2. A comparison of the fit, retention, fracture resistance, surface roughness, biocompatibility, and aesthetics of the base in conventional dentures and CAD/CAM-fabricated dentures.

The null hypothesis is that digital dentures show better results in all the investigated variables.

## 2. Materials and Methods

A search was conducted for articles related to the fabrication of both conventional and digital complete mucosa-supported dental prostheses and their mechanical and aesthetic properties. The databases used were PubMed/MEDLINE, Cochrane Library, Scielo and Embase, with the keywords digital, removable complete dental prostheses, CAD/CAM, and complete denture. Additionally, the Boolean operator OR was used in the search equation [(Removable complete dental prostheses) OR (digital)]. The inclusion and exclusion criteria used were as follows (Table 1):

## 3. Results

**Figure 1:**
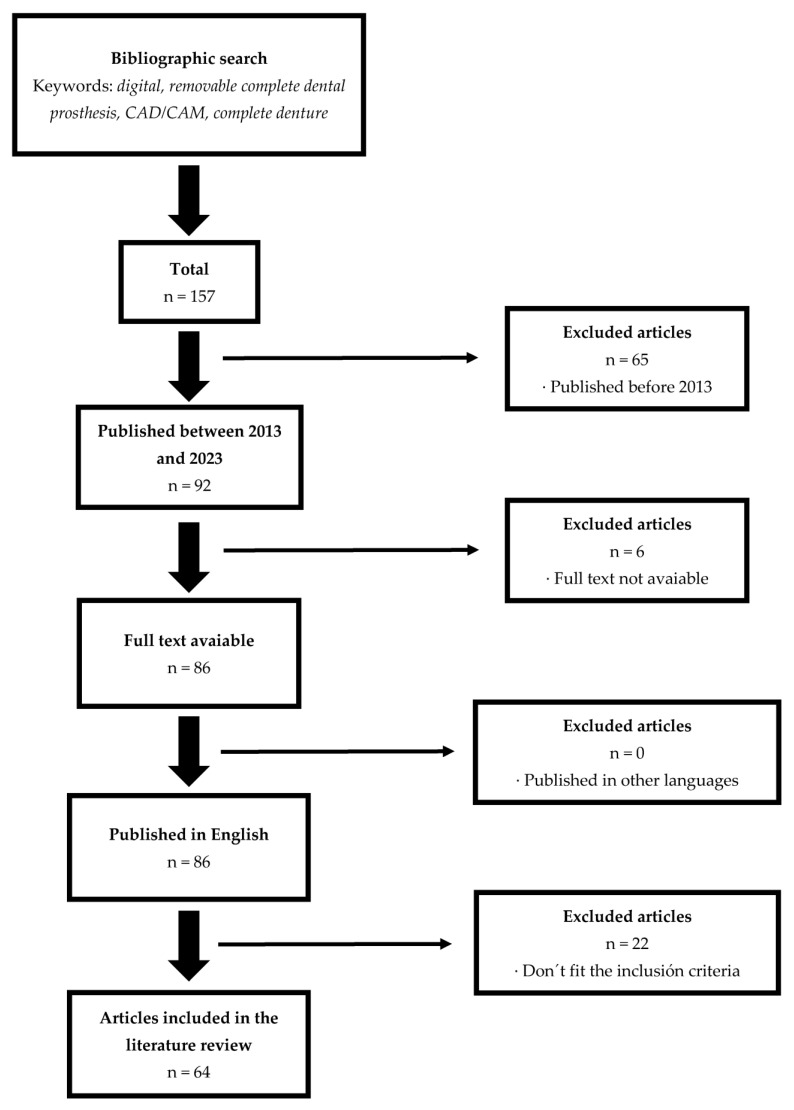
Flowchart of the search performed.

**Figure 2:**
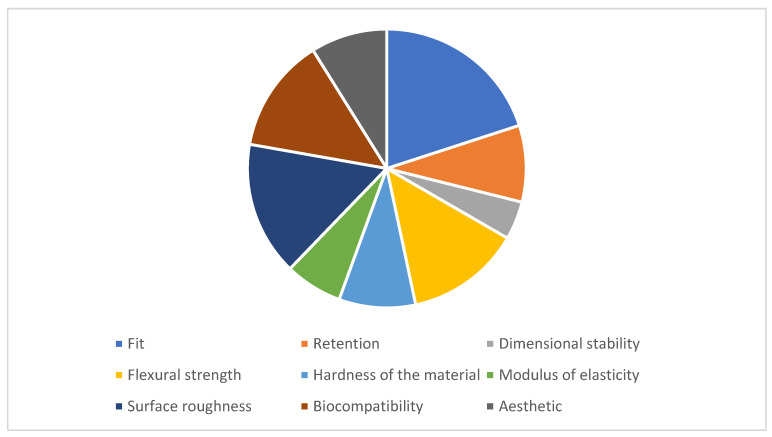
Analyzed variables.

In the following flowchart (Figure 1), the conducted search and its outcomes are depicted:

A total of 40 articles were analyzed in the conducted review. All articles were published after 2017, and they were all clinical trials (in vitro or in vivo). Among these, nine publications focused on analyzing base fit, four discussed retention, and two addressed dimensional stability. Regarding material properties, six articles discussed flexural strength, three covered material hardness, three discussed the modulus of elasticity, and seven articles examined material roughness and porosity. Finally, six publications discussed material biocompatibility, and four of them explored topics related to aesthetics and color stability. Among the analyzed articles, 40 of them discussed milled prosthetics, and 10 discussed printed prosthetics (comparing them with prosthetics manufactured using conventional methods) (Figure 2).

## 4. Discussion

In most cases, patients prefer digital complete dentures over the conventional dentures they had previously. One of the main factors influencing this preference is the reduced treatment time, which decreases from five appointments to two appointments [20,37]. However, according to Ohara et al. [11], patient satisfaction with conventional dentures is significantly higher than with digital dentures, and within the digital dentures category, patients prefer milled dentures over those manufactures using 3D printing technology. This is partly because printed dentures require a minimum thickness of 2.5 mm to ensure base strength, while milled and conventional dentures only require a thickness of 1.4 mm, resulting in greater comfort for patients during speech. Additionally, the teeth used in milled and conventional dentures are made of a harder resin compared to the resin used in 3D printers, resulting in fewer color changes, better resemblance to natural teeth, and easier maintenance. However, teeth used in conventional dentures still exhibit better color stability than milled dentures. Another advantage of conventional dentures is the ease of placing teeth in different axes, especially in the anterior region. In milled dentures, as the teeth are milled from a single piece, it is more challenging to change the angulation of each tooth individually. Patients also prefer conventional dentures due to their better stability, which can be attributed to the peripheral seal of the denture and stable occlusal contacts, which are not as effective in digital dentures [11].

Some researchers attribute patient dissatisfaction to psychological factors and even differences in perspective between the clinician and the patient. In cases where a patient has complex oral characteristics, the professional may assess a prosthesis as adequate, which under normal circumstances may not meet the patient´s expectations. Consequently, the patient may end up with an unsatisfactory prosthesis. Alfadda S. [1], after studying all the aforementioned variables, concludes that the lack of stability and retention in mandibular dentures has the most significant impact on the acceptance of complete dentures. Several studies have shown that the lack of stability in the lower denture leads to difficulties in chewing and an increase in the mobility of the upper denture [1].

Numerous studies (Table 2) have demonstrated better retention in digital dentures compared to conventional dentures, which exhibit less dimensional stability mainly due to the absence of polymerization shrinkage in the denture base [25,30,35,38,39,40,41].

The shrinkage of PMMA can cause volumetric distortion in the denture base, which has traditionally been counteracted by hydrating the denture with water. The expansion resulting from this hydration offsets the polymerization shrinkage, depending on the amount of free monomer. In the study by AlHelal et al. [25], both conventional and digital dentures were fabricated, and both were immediately immersed in water after their fabrication. The results showed a significant increase in retention in the digital dentures compared to the conventional ones. Thus, hydration may not be sufficient to compensate for the polymerization shrinkage that occurs in conventional dentures. This difference between the two groups could be explained by the higher density of the milled denture bases, resulting in greater dimensional stability and reducing the influence of hydration on retention [25]. However, in the research by Srinivasan et al. [44], which compared the fit of dentures fabricated using two conventional techniques (flasking and injection molding of resin) and milling, all three techniques demonstrated clinically acceptable fit with no significant differences prior to hydration. After hydration with artificial saliva, the dentures fabricated using flasking improved their fit significantly, contrary to the findings of the study by AlHelal et al. [25,44].

It is believed that 3D printing resins, which are applied in liquid from to the model, may lead to a better fit, but currently, there is not enough evidence to support this. However, the need to polymerize these resins after their conformation can be a disadvantage in terms of fit, due to the polymerization shrinkage these resins undergo, leading to detachment of the palate. This compromises the suction effect of the denture. This phenomenon also occurs in conventional complete dentures made of thermopolymerizable resin and can be compensated for by creating a post-dam in the denture or hydrating the denture base with water; the resulting expansion due to hydration can counteract the deformation produced via polymerization, depending on the amount of free monomer [18,25]. In milled dentures, on the other hand, polymerization takes place before the denture is formed. However, more research is needed to determine if, in this case, a post-dam would also be necessary to achieve this suction effect [18]. The additive manufacturing uses resins that, once processed, require a final photopolymerization. Until this photopolymerization is performed, the resins may undergo polymerization shrinkage and compromise their mechanical properties. Additionally, the removal of the denture from the 3D printer occurs before the final polymerization, which can result in denture deformation. Invariably, an unpolymerized resin layer, the inhibited layer, remains in the finished denture, which must be removed with an appropriate solvent. Although all these situations may result in less fit and dimensional stability of the printed dentures compared to the milled ones, the additive manufacturing has many advantages such as precision, less material waste, and more economical machinery. Today, 3D printers are cheaper and easier to transport than milling machines, making them more accessible for private clinics and dental laboratories, eliminating the time and costs of shipping [18,47]. However, some authors such as Lee et al. [46] or Hwang et al. [43] obtained results in their studies showing that printed dentures had better fit than milled dentures and conventional dentures [42,43].

Faty et al. [7] concluded that PMMA pre-polymerized denture bases showed better adaptation to the supporting tissues tan those made of conventionally polymerized PMMA, similar to the findings of Goodacre et al. [26] and Hsu et al. [48], who determined that digital dentures exhibit superior adaptation compared to dentures fabricated via any conventional method [7,26,48]. This is attributed to the reduced occurrence of dimensional changes during polymerization in the case of digital dentures. Additionally, pre-polymerized PMMA provides better retention due to its lower polymerization shrinkage. However, a disadvantage could be its higher cost and increased material waste. Faty et al. [7] also found significant differences in the retention of the three types of dentures analyzed: conventional, milled, and printed dentures. Furthermore, they demonstrated that milled dentures had higher retention than conventional dentures [7].

The fit of the denture base to the supporting tissues is essential for proper retention, stability, and support of complete dentures. Minimal distortion during the denture fabrication process is crucial to achieve a correct adaptation to the mucosa. The degree of distortion depends on both the thickness and material used, as well as the fabrication technique. In the milling of denture bases, dimensional deformation due to polymerization is avoided, and a smoother surface, meaning lower surface roughness, is achieved. In the study by Faty et al. [7], milled dentures showed better fit, greater adaptation, fewer dimensional changes, and higher precision during the fabrication process. In the case of conventional dentures, the complexity of the procedure, the required time, and the deformation due to PMMA polymerization may reduce the degree of adaptation of the denture base [6,7].

The quality of the impression, whether obtained conventionally with materials like hydrocolloids or polyvinyl siloxanes or digitally trough scanning, is a determining factor in the final fit of the denture base. Information for CAD/CAM processing can be acquired either extraorally by scanning an impression or a model of the dental arch, or intraorally, by directly scanning the dental arch. There are different systems: mechanical digitalization, based on touch, and optical digitalization, which utilizes CBCT, laser, or light-emitting diode scanners [14]. In 2007, Quaas et al. [49] studied the measurement uncertainty and accuracy of data obtained from mechanical digitalization and concluded that the measurement uncertainty was low, while the accuracy was high. However, they ruled out this method for scanning impressions with flexible materials, as the physical contact of the scanner with the impression could deform it and increase imprecision [14,49]. In 2012, Goodacre et al. [12] introduced a technique to obtain definitive impressions of edentulous maxillary and mandibular arches, which would later be scanned. They also described how to record the neutral zona, the position of the maxillary and mandibular anterior teeth, palatal morphology, VDO, and interocclusal relationship, and added this data to the scanned impressions to fabricate the denture base trough milling. Additionally, it is essential to consider that saliva on soft tissues and their movements can influence the quality of digital impressions [7,12,14].

Flexural strength (Table 3) is considered a reflection of the resistance and stiffness of the studied material and serves to evaluate the quality of material polymerization [50]. Although PMMA acrylic resins are the most used materials in the fabrication of both digital and conventional dentures, they are susceptible to fractures due to the material´s brittleness when subjected to impacts. Thermopolymerizable resins used in the fabrication of conventional dentures exhibit good physical and mechanical properties, although some drawbacks related to polymerization have been described, such as high porosity, potential for cracking, and volumetric and linear shrinkage [51]. On the other hand, pre-polymerized resins used in milled dentures exhibit higher flexural strength. These resin discs are polymerized under high temperature and pressure, leading to the formation of longer polymer chains, resulting in lower residual monomer values and minimal porosity. Additionally, the polymerization process leads to a reduction in the intermolecular distance of the resin, which could explain the improved mechanical behavior of pre-polymerized PMMA [51].

Materials with a high modulus of elasticity are more resistant to elastic deformation, allowing the fabrication of thinner denture bases. Becerra et al. [54] and Iwaki et al. [55] determined that digital dentures exhibited a higher modulus of elasticity compared to conventional dentures [54,55]. The main advantage of this is that a denture base resistant to plastic deformation provides greater occlusal stability [51]. In several studies by Al-Dwairi et al. [50,52] (2019 and 2020), digital dentures showed higher flexural strength and greater surface hardness, indicating a higher modulus of elasticity than conventional dentures, which was also confirmed in the study by Alp et al. This could be attributed to a lower monomer content in digital dentures, reducing the plasticity of PMMA [50,52,53]. However, in the study by Perea-Lowery et al. [51], no significant differences in hardness were found between the two groups [6,51]. Prpić V et al. [36] confirmed the results of Perea-Lowery et al. [51], but pointed out that the different results were not solely due to different polymerization methods, but could also be influenced by the use of different materials from different manufacturers [6,36,51].

Many authors such as Angelara et al. [56] and Arslan et al. [16] state that the flexural strength of pre-polymerized PMMA (used in milled dentures) is higher than conventionally polymerized PMMA due to conditions under which polymerization occurs (for pre-polymerized PMMA, high pressures and temperatures), the homogeneity of the denture base and its higher density, minimal contraction that takes place, and the reduced amount of pores and free monomer [16,56]. Choi et al. [57] and Srinivasan et al. [13] reported that conventionally polymerized PMMA had higher toughness than pre-polymerized PMMA, meaning it could absorb more force before fracturing, attributed to the amount of free monomer in the material: higher monomer content resulted in greater material plasticity and, therefore, higher toughness [6,18,36,57]. Over time, pre-polymerized PMMA exhibits less swelling and deterioration than conventional PMMA due to lower water absorption and less difference in thermal expansion between the denture base and artificial teeth, resulting in minimal differences in terms of toughness [6].

Prosthetic stomatitis is an inflammation of the palatal mucosa covered by the denture caused by the accumulation of *Candida albicans*. A smooth surface is important in any restoration to reduce the formation of a biofilm. Milled denture bases have lower porosity, while conventional dentures, even after final polishing, are associated with a higher presence of *Candida* [37]. Numerous researchers such as Al-Dwairi et al. [52], Klaiber et al. [34], Chang et al. [58], Murat et al. [59], or Al-Fouzan et al. [60] (Table 4) found that the surface roughness of PMMA denture bases in conventional dentures was higher than in milled PMMA. The reduced number and size of pores are mainly due to a lower amount of residual monomer and the polymerization technique, which gives digital dentures better properties, such as reduced microbial adhesion to the surface, less plaque retention, and fewer surface alterations. However, other authors like Arslan et al. [16] did not find significant differences in surface roughness between both types of dentures. This discrepancy in the results may be due to factors such as different water solubility, hardness, microstructure, and chemical configuration of the studied materials [6,16,34,52,58,59,60]. In the case of printed dentures, Srinivasan et al. [13] determined that the degree of surface roughness was similar to milled dentures. However, using resins and 3D printers not recommended by the manufacturer may negatively influence this aspect, significantly increasing the roughness of the denture base [18].

The biocompatibility (Table 5) of dentures largely depends on the amount of residual free monomer released by the resin after polymerization. Ayman et al. [17] observed that the release of free monomer was higher in the thermopolymerizable resins used in the fabrication of conventional dentures, mainly due to the pre-polymerization of the discs used for milling the dentures [17]. According to Engler et al. [61], the monomer release depends on the material used, so it will be different depending on the manufacturing method used. In the case of conventional dentures, the initial monomer release is considerably higher than in digital dentures but remains more stable over time, while pre-polymerized PMMA increases the monomer release over the days [61]. However, other authors such as Steinmassl et al. [45] concluded that although digital dentures had a lower amount of free monomer than conventional dentures, the differences were not significant [45].

Another influential factor in the biocompatibility of dentures is microbial adhesion (Table 5), closely related to the previously mentioned surface roughness. Both Al-Fouzan et al. [60] and Murat et al. [59] found that microbial adhesion, mainly by *Candida albicans*, was higher in conventional dentures due to increased surface roughness [59,60]. In terms of overall biocompatibility, both milled and 3D-printed dentures showed good compatibility with oral tissues, with no significant differences between them [1].

Regarding aesthetics *(*Table 6), the most decisive factor is the color stability of both the denture base and the used teeth. This variable is inversely related to surface roughness: the higher the surface roughness, the lower the color stability. Authors such as Dayan et al. [62] and Iwaki et al. [55] concluded that the resins used in milled dentures showed fewer color changes compared to other resins [37,50]. Gruber et al. [63] found no significant differences between conventional dentures and milled dentures, with the 3D-printed resins showing the most color changes [51]. However, some studies, like the one by Alp et al. [53], confirmed that the color changes observed in any type of denture were within clinically acceptable ranges [39].

The null hypothesis was accepted, as digital prosthetics demonstrated better performance than conventional prosthetics. However, the review presents numerous limitations such as the different manufacturing methods, the small sample size used in the consulted studies, or the need to consult more articles.

The future of dentistry is being explored through the possibilities offered by digital systems. The advances made possible by these systems are evident today. Concerning the topic of complete prosthetics produced using digital systems, there is still a long way to go, largely due to the need to research improvements in materials and the necessity to enhance and reduce the cost of the equipment that facilitates their fabrication. The use of digital systems for prostheses allows the dentist to intervene and modify parameters to meet the patient´s needs. Furthermore, it constitutes an important marketing tool for convincing the patient (previews and smile design). However, it comes with certain drawbacks, such as a higher production cost, the need for equipment investment, and a steep learning curve to achieve satisfactory results. Undoubtedly, the future of prosthodontics lies in applying all the technological advancements at our disposal, which are already demonstrating their ability to address the issues associated with conventional methods. 

## 5. Conclusions

As a result of the conducted review and in accordance with the specific objectives outlined, the following conclusions were drawn:

1. Regarding fit, retention, fracture resistance, surface roughness, biocompatibility, and aesthetics, digital prostheses, particularly the milled ones, have demonstrated superior performance.
2. The planning process of digital prostheses, with its advantage of preserving all data and prosthetic designs, except anatomical registers, facilitates quick retrieval at any time.

## Figures and Tables

**Table 1:** Inclusion and exclusion criteria.

Inclusion Criteria	Exclusion Criteria
Articles published from 2012 onwards	Articles published before 2012, except for the Quaas study (2007), which was included due to its significance in the evolution of obtaining patient arch models through CAD/CAM
Articles available in full text Articles focused on human subjects or in vitro trial Systematic reviews and clinical trials were included Articles published in English	Articles whose content was not available Articles whose subject of study was animal species Other types of studies that were not clinical trials or systematic review Articles published in languages other than English Publications that did not align with the aim of the review

**Table 2:** Analysis of prosthetic fit and retention.

Author	Type of Study	Year	Studied Variable	Manufacturing Method	Sample Size	Conclusions
Tasaka et al. [42]	Clinical trial	2019	Fit and retention	Conventional method (flasking)	1	The prostheses manufactured using 3D printing demonstrated better fit and, therefore, better retention than conventional prostheses.
				Digital method (3D printing)	1	
Hwang et al. [43]	Clinical trial	2019	Fit	Conventional method (flasking)	10	The adaptation to the tissues and fit was higher in the printed prostheses than in the other two groups studied.
				Digital method (milling)	10	
				Digital method (3D printing)	10	
Srinivasan et al. [44]	Clinical trial	2017	Fit	Conventional method (flasking)	11	Initially, all three techniques demonstrated acceptable fit, and there were no significant differences between them. However, after hydrating the prostheses with saliva, the fit of the prostheses fabricated using conventional flasking improved significantly.
				Conventional method (resin injection)	11	
				Digital method (milling)	11	
Steinmassl et al. [45]	Clinical trial	2018	Fit	Conventional method (flasking)	5	The digital prostheses exhibit better fit than conventional prostheses.
				Digital method (milling)	20	
Lee et al. [46]	Clinical trial	2019	Fit	Conventional method (resin injection)	10	The prostheses fabricated via 3D printing exhibited better fit than the milled and conventional prostheses.
				Digital method (milling)	10	
				Digital method (3D printing)	10	
Faty et al. [7]	Clinical trial	2021	Fit and retention	Conventional method	24	The milled prostheses demonstrated better fit and retention than the other two groups. The printed prostheses showed better fit but similar retention to the conventional prostheses.
				Digital method (milling)	24	
				Digital method (3D printing)	24	
Goodacre et al. [26]	Clinical trial	2016	Fit	Conventional method (flasking)	10	The digital prostheses showed greater adaptation to the supporting tissues than any of the conventional methods.
				Conventional method (resin injection)	10	
				Conventional method (self-curing resin)	10	
				Digital method (milling)	10	
McLaughlin et al. [40]	Clinical trial	2019	Fit	Conventional method (flasking)	27	The conventional prostheses fabricated using flasking exhibited a significantly lower fit than the other two groups.
				Conventional method (resin injection)	27	
				Digital method (milling)	27	
AlHelal et al. [25]	Clinical trial	2017	Retention	Conventional method (flasking)	20	The retention of digital prostheses was significantly higher than that of conventional prostheses.
				Digital method (milling)	20	
Einarsdottir et al. [39]	Clinical trial	2020	Dimensional stability	Conventional method (resin injection)	15	Conventional prostheses have lower dimensional stability than the digitally milled prostheses.
				Digital method (milling)	15	
Wemken et al. [47]	Clinical trial	2020	Dimensional stability	Conventional method (resin injection)	16	The milled prostheses demonstrated the highest dimensional stability, followed by the conventional prostheses, and finally, the printed prostheses.
				Digital method (milling)	16	
				Digital method (3D printing)	16	
AlRumaih et al. [38]	Clinical trial	2018	Retention	Conventional method (flasking)	20	The milled prostheses showed higher retention than the conventional prostheses.
				Digital method (milling)	20	
Hsu et al. [48]	Clinical trial	2020	Fit	Conventional method (flasking)	10	Both the conventional and milled prostheses exhibited better fit than the printed prostheses, with the milled prostheses demonstrating the best adaptation to the supporting tissues.
				Conventional method (resin injection)	10	
				Digital method (milling)	20	
				Digital method (3D printing)	20	

**Table 3:** Analysis of the flexural strength, modulus of elasticity, and hardness of the prosthetic base.

Author	Type of Study	Year	Studied Variable	Manufacturing Method	Sample Size	Conclusions
Al-Dwairi et al. [50]	Clinical trial	2020	Flexural strength	Conventional method (flasking)	15	The flexural strength (rigidity) is higher in the digital prostheses
				Digital method (milling)	30	
Al-Dwairi et al. [52]	Clinical trial	2019	Superficial hardness	Conventional method (flasking)	15	The surface hardness is higher in the digital prostheses than in the conventional prostheses
				Digital method (milling)	30	
Perea-Lowery et al. [51]	Clinical trial	2021	Flexural strength	Conventional method (flasking)	8	Digital prostheses exhibit higher flexural strength than conventional prostheses, although the differences found were not significant
				Conventional method (self-curing resin)	8	
				Digital method (milling)	24	
Srinivasan et al. [30]	Clinical trail	2021	Modulus of elasticity and superficial hardness	Digital method (milling)	10	The milled prostheses showed higher surface hardness, higher modulus of elasticity and greater force absorption capacity than the printed prostheses
				Digital method (3D printing)	20	
Alp et al. [53]	Clinical trial	2019	Flexural strength	Conventional method (flasking)	15	Digital prostheses exhibited greater flexural strength than conventional prostheses
				Conventional method (self-curing resin)	15	
				Digital method (milling)	45	
Arslan et al. [16]	Clinical trial	2018	Flexural strength	Conventional method (flasking)	10	The digital prostheses exhibited greater flexural strength than the conventional prostheses
				Digital method (milling)	30	
Prpić et al. [36]	Clinical trial	2020	Flexural strength and superficial hardness	Conventional method (flasking)	160	The flexural strength is higher in the milled prostheses, as well as the hardness (with non-significant differences)
				Digital method (milling)	160	
				Digital method (3D printing)	160	
				Digital method (milling)	30	
Becerra et al. [54]	Clinical trial	2021	Modulus of elasticity and mechanical strength	Conventional method (flasking)	30	The digital prostheses demonstrated higher mechanical strength and a lower modulus of elasticity compared to the conventional prostheses
				Conventional method (resin injection)	30	
				Digital method (milling)	30	
Iwaki et al. [55]	Clinical trial	2020	Modulus of elasticity	Conventional method (flasking)	5	The digital prostheses exhibited a higher modulus of elasticity than the conventional prostheses
				Digital method (milling)	5	
Angelara et al. [56]	Clinical trial	2023	Flexural strength	Conventional method	20	The digital prostheses have greater flexural strength than the conventional prostheses
				Digital method (milling)	40	

**Table 4:** Analysis of the surface roughness of the prosthetic base.

Author	Type of Study	Year	Studied Variable	Manufacturing Method	Sample Size	Conclusions
Al-Dwairi et al. [52]	Clinical trial	2019	Surface roughness	Conventional method (flasking)	15	The surface roughness is higher in conventional prostheses than in digital prostheses
				Digital method (milling)	30	
Srinivasan et al. [18]	Clinical trial	2021	Surface roughness	Digital method (milling)	10	Both types of prostheses exhibit similar surface roughness, although prostheses printed with resins not recommended by the manufacturer may result in rougher surfaces
				Digital method (3D printing)	20	
Chang et al. [58]	Clinical trial	2021	Surface roughness	Conventional method (self-curing resins)	10	The digital prostheses showed lower surface roughness than the prostheses made with self-curing resins
				Digital method (milling)	5	
Arslan et al. [16]	Clinical trial	2018	Surface roughness	Conventional method (flasking)	10	Conventional prostheses have higher surface roughness than digital prostheses, but the differences are not significant
				Digital method (milling)	30	
Murat et al. [59]	Clinical trial	2019	Surface roughness	Conventional method (flasking)	10	The digital prostheses exhibited lower surface roughness than the conventional prostheses
				Digital method (milling)	30	
Klaiber et al. [34]	Clinical trial	2021	Surface roughness	Conventional method	60	The conventional prostheses have higher surface roughness than the digital prostheses
				Digital method (milling)	60	
Al-Fouzan et al. [60]	Clinical trial	2017	Surface roughness	Conventional method (flasking)	10	The surface roughness in conventional prostheses was significantly higher than in digital prostheses
				Digital method (milling)	10	

**Table 5:** Analysis of the biocompatibility of the prosthetic base.

Author	Type of Study	Year	Studied Variable	Manufacturing Method	Sample Size	Conclusions
Al-Fouzan et al. [60]	Clinical trial	2017	Microbial adhesion	Conventional method (flasking)	10	The digital prostheses exhibited lower superficial adhesion of *Candida albicans* compared to conventional prostheses
				Digital method (milling)	10	
Murat et al. [59]	Clinical trial	2019	Microbial adhesion	Conventional method (flasking)	10	*Candida albicans* adhesion was significantly higher in conventional prostheses compared to digital prostheses
				Digital method (milling)	30	
Srinivasan et al. [18]	Clinical trial	2021	Biocompatibility	Digital method (milling)	18	Both methods demonstrated good compatibility with oral tissues, with no significant differences observed between them
				Digital method (3D printing)	18	
Engler et al. [61]	Clinical trial	2020	Release of residual monomer	Conventional method (flasking)	40	The monomer release depends on the material used, remaining more stable over time in the case of material used in the fabrication of conventional prostheses
				Digital method (milling)	320	
Ayman et al. [17]	Clinical trial	2017	Release of residual monomer	Conventional method (flasking)	15	The resins used for milling digital prostheses have a lower content of free monomers compared to thermopolymerizable resins
				Digital method (milling)	15	
Steinmassl et al. [45]	Clinical trial	2017	Release of residual monomer	Conventional method (flasking)	10	Conventional prostheses exhibited a slightly higher monomer release compared to digital prostheses, but the difference was not significant
				Digital method (milling)	40	

**Table 6:** Aesthetic analysis of the prostheses.

Author	Type of Study	Year	Studied Variable	Manufacturing Method	Sample Size	Conclusions
Dayan et al. [62]	Clinical trial	2019	Color stability	Conventional method (thermopolymerizable, photocurable, and self-curing resins)	45	The color stability in the resins used for milling prostheses is superior to any other type of resins.
				Digital method (milling)	15	
Gruber et al. [63]	Clinical trial	2021	Color stability	Conventional method (flasking)	8	There were no differences in color stability between conventional resins and those used in milling. However, the 3D printing resins showed significant changes in coloration compared to the other two groups.
				Digital method (milling)	28	
				Digital method (3D printing)	8	
Alp et al. [53]	Clinical trial	2019	Color stability	Conventional method (flasking)	6	The color changes (due to coffee exposure) were imperceptible in all groups.
				Digital method (milling)	18	
Iwaki et al. [55]	Clinical trial	2020	Color stability	Conventional method (flasking)	3	The milled prostheses exhibited greater color stability than the conventional prostheses.
				Digital method (milling)	3	
